# Impact of sleep quality on post‐stroke anxiety in stroke patients

**DOI:** 10.1002/brb3.1716

**Published:** 2020-11-03

**Authors:** Meijuan Xiao, Guiqian Huang, Liang Feng, Xiaoqian Luan, Qiongzhang Wang, Wenwei Ren, Siyan Chen, Jincai He

**Affiliations:** ^1^ Department of Neurology The First Affiliated Hospital of Wenzhou Medical University Wenzhou China

**Keywords:** income, insomnia, Pittsburgh Sleep Quality Index, post‐stroke anxiety, sleep quality

## Abstract

**Objective:**

To explore whether poor sleep is associated with post‐stroke anxiety (PSA) in Chinese patients with acute ischemic stroke (AIS) and to verify whether poor sleep is a predictor of PSA.

**Methods:**

A total of 327 patients with AIS were enrolled and followed up for 1 month. Sleep quality within 1 month before stroke was evaluated using the Pittsburgh Sleep Quality Index (PSQI) at admission. The patients were divided into the poor sleep group (PSQI > 7, *n* = 76) and good sleep group (PSQI ≤ 7, *n* = 251). One month after stroke, patients with obvious anxiety symptoms and a Hamilton Anxiety Scale score >7 were diagnosed with PSA.

**Results:**

Eighty‐seven patients (26.6%) were diagnosed with PSA. Compared to the good sleep quality group, the incidence of PSA in patients with poor sleep quality was higher (42.1% vs. 21.9%, *p* = .001). Poor sleep quality is more common in patients with PSA (35.6% vs. 18.8%, *p* = .001). A logistic regression analysis indicated that poor sleep quality was significantly associated with PSA (OR: 2.265, 95% CI: 1.262–4.067, *p* = .003). After adjusting for conventional and identified risk factors, poor sleep quality was found to be independently associated with PSA (OR: 2.676, 95% CI: 1.451–4.936, *p* = .001).

**Conclusions:**

Poor sleep quality before stroke was associated with PSA and may be an independent risk factor of PSA 1 month after AIS onset.

## Graphical abstract

We found that the incidence rate of poor sleep quality is significantly higher in PSA patients than in non‐PSA patients. Poor sleep quality before stroke is independently associated with the development of PSA, even after adjustment for several conventional confounders. These findings suggested poor sleep quality before stroke could provide important predictive information for anxiety after acute ischemic stroke.

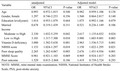

## 1 INTRODUCTION

Stroke is defined as a sudden loss of focal cerebral function. This process lasts for 24 hr or more and is thought to be caused by an inadequate blood supply to some parts of the brain (ischemic stroke), or spontaneous hemorrhage of brain substance (primary intracerebral hemorrhage), where brain imaging was normal or showed evidence of recent ischemia or hemorrhage. Conversely, focal arterial ischemia with transient symptoms (lasting <24 hr) and without any evidence of infarction according to pathological or imaging examinations should be considered a TIA (transient ischemic attack; Sacco et al., 2013). Stroke is the second most common cause of death worldwide as well as the leading cause of long‐term disability (Tsai, Thomas, & Sudlow, 2013). Mood and emotional disturbances are frequent symptoms in stroke survivors (Hackett, Köhler, O'Brien, & Mead, 2014), including post‐stroke depression (PSD), post‐stroke anxiety (PSA), post‐stroke emotional incontinence (PSEI), post‐stroke anger proneness (PSAP), and post‐stroke fatigue (PSF; Kim, 2016). Previous studies have found that PSA is closely associated with PSD (Campbell Burton et al., 2013), and three‐quarters of the anxious patients had comorbid major or minor depression (Castillo, Schultz, & Robinson, 1995). The core symptoms of PSA are excessive anxiousness or worry, and difficulty in controlling worries. In addition to these symptoms, a diagnosis of anxiety requires three or more of the following: restlessness, decreased energy, poor concentration, irritation, nervous tension, and insomnia (American Psychiatric Association, 2013). Patients with PSA do not have a previous history of anxiety disorder prior to the onset of the stroke and can be considered an emotional complication after stroke. The combined rate of anxiety by time after stroke was as follows: 20% within 1 month after stroke onset; 23% 1–5 months after stroke onset; and 24% six or more months after stroke onset (Campbell Burton et al., 2013). Despite the high prevalence of anxiety after stroke, the understanding of PSA is still limited (Cumming, Blomstrand, Skoog, & Linden, 2016; Kim, 2016; Liu, Cai, Zhang, Zhu, & He, 2018).

Anxiety‐related neural circuits span a wide range of brain structures, including subcortical white matter and the limbic system (Allsop, Vander Weele, Wichmann, & Tye, 2014; Westlye, Bjørnebekk, Grydeland, Fjell, & Walhovd, 2011). However, the pathophysiological mechanisms underlying the development of PSA remain unclear. Currently, some studies have found that PSA is associated with greater dependence in daily activities, higher mortality and poorer overall quality of life (Broomfield, Quinn, Abdul‐Rahim, Walters, & Evans, 2014; Campbell Burton et al., 2013; Li et al., 2019). Therefore, identifying the risk factors for PSA is urgent and necessary. Previous studies have shown that age, gender, inability to work, depression, smoking, stroke severity, low levels of serum 25‐hydroxyvitamin D [25(OH)D ≤ 38.48 nmol/L], stroke areas, previous history of insomnia, and low socioeconomic status are associated with anxiety disorders (Ayerbe, Ayis, Crichton, Wolfe, & Rudd, 2014; Bushnell et al., 2014; Kuchcinski et al., 2017; Leppävuori, Pohjasvaara, Vataja, Kaste, & Erkinjuntti, 2003; Li et al., 2019; Thayabaranathan et al., 2018; Wu et al., 2016). However, no consensus has been reached regarding the risk factors of anxiety in stroke patients.

Anxiety is one of the most common mental disorders, and an epidemiological study has shown that insomnia and anxiety disorders are closely related (Ohayon, 2002). A systematic review concluded that the relationship between insomnia and anxiety is bidirectional (Alvaro, Roberts, & Harris, 2013). Insomnia has been found to be a risk factor of anxiety in the general population as well as in pre‐ and post‐menopausal women and adolescents (Friedman, Brooks, Bliwise, & Yesavage, 1993; Johnson, Roth, & Breslau, 2006; Neckelmann, Mykletun, & Dahl, 2007; Osnes, Roaldset, Follestad, & Eberhard‐Gran, 2019; Swanson, Pickett, Flynn, & Armitage, 2011; Terauchi et al., 2012). Similarly, epidemiological studies have shown a relationship between sleep quality and anxiety. Previous studies have shown that sleep disorders, difficulty in falling asleep, and frequent sleep deficits increase the risk of anxiety. Poor sleep quality is associated with anxiety, whether in children and adolescents, college students, pregnant women, or the elderly (Adams & Kisler, 2013; Brown et al., 2018; Volkovich, Tikotzky, & Manber, 2016; Xiong et al., 2019). A study of 3,987 Chinese elderly, aged 60 years or more, published in 2020, suggests that compared with those with good sleep quality, the OR (95% CI) of anxiety for those with poor sleep quality was 5.12 (3.88–6.77) (Shi et al., 2020). Post‐stroke insomnia (PSI) has a significant incidence in the acute phase of stroke (Sterr et al., 2018). Up to 70% of the patients with acute stroke have sleep disorders including excessive daytime sleepiness, insomnia, hypersomnia, and fatigue (Pasic, Smajlovic, Dostovic, Kojic, & Selmanovic, 2011). Nevertheless, the association between sleep quality before stroke and PSA in patients with acute ischemic stroke (AIS) has not been elucidated.

The present study was conducted to explore the relationship between sleep quality at admission and PSA in Chinese patients 1 month after the stroke.

## 2 MATERIALS AND METHODS

### 2.1 Subjects

**FIGURE 1:**
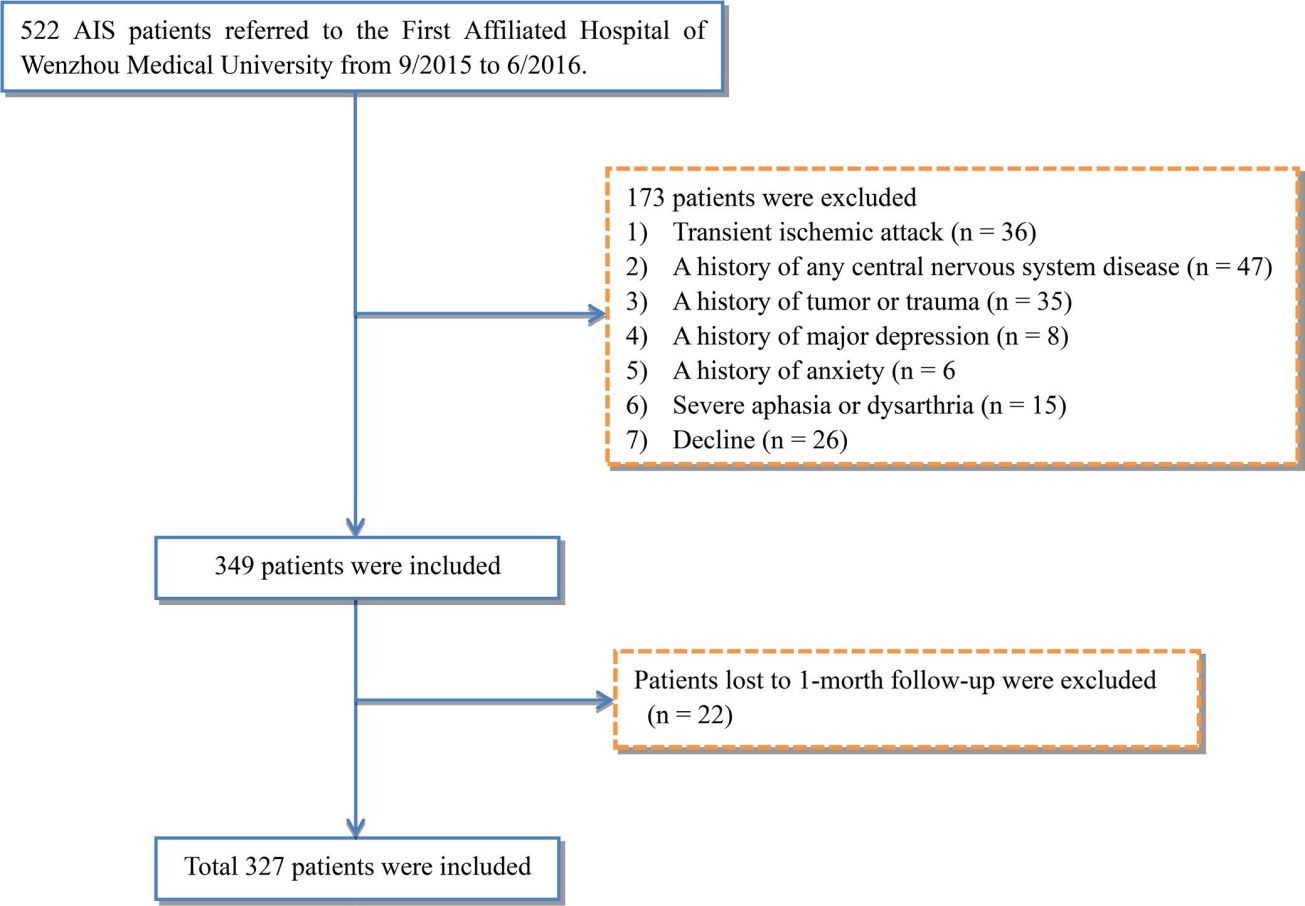
Study flow diagram. AIS, acute ischemic stroke

The patients' data from September 2015 to June 2016 were collected from the stroke unit of the First Affiliated Hospital of Wenzhou Medical University. The inclusion criteria were as follows: (a) Chinese ethnicity, (b) 18–80 years of age, (c) suffered acute stroke within 3 days before admission, (d) CT and/or MRI supported diagnosis of AIS, and (e) informed consent from the patient. A total of 412 AIS patients were screened. From them, 173 patients were excluded (36 for TIA, 47 for history of nervous system disease, 35 for history of tumor or trauma, eight for a history of depression, six for a history of anxiety, 15 for severe aphasia, and 26 for decline). Thus, 349 patients were enrolled in the present study. One month after AIS onset, follow‐up could not be done with 22 patients. The final analysis included 327 patients (Figure 1).

This study was conducted following the Helsinki Declaration (World Medical Association, 2013). Participation in the study was voluntary, and written informed consent was obtained. The participants could withdraw their consent without any explanation at any time during the study. This study was approved by the ethical committee of The First Affiliated Hospital of Wenzhou Medical University (Wenzhou, China).

### 2.2 Data collection

The patients' demographic information, including age, gender, education (none, i.e., 0 year; primary, i.e., 6 years or less; secondary, i.e., 7–9 years; higher, i.e., more than 9 years), marital status (married or single), and income (low annual household income, i.e., 12,000 yuan or less; moderate annual household income, i.e., 12,000 yuan – 60,000 yuan; high annual household income, i.e., more than 60,000 yuan), was assessed. The baseline vascular risk factors, including hypertension (a history of hypertension or anti‐hypertensive medication), diabetes (a history of diabetes or anti‐diabetes medication), cigarette smoking, current alcohol consumption, and coronary artery disease, were evaluated. The patient's levels of serum 25‐hydroxyvitamin D [25(OH)D] and HbA1c on the second day after admission were retrieved. Vitamin D deficiency was defined as the level of 25(OH)D being ≤38.48 nmol/L following the previous study (Wu et al., 2016). HbA1c was assessed using ion‐exchange chromatography. The evaluation of stroke severity was determined using the National Institutes of Health Stroke Scale (NIHSS; Goldstein & Samsa, 1997). Sleep quality was evaluated at admission using the Pittsburgh Sleep Quality Index (PSQI; Buysse, Reynolds, Monk, Berman, & Kupfer, 1989). Functional outcome was measured using the Barthel Index (BI). Cognition function was determined using the Mini‐Mental State Examination (MMSE) 1 month after the stroke. Poor functional outcome was defined as a BI score of 60 or less. Moreover, all patients were screened for anxiety symptoms using the 17‐item Hamilton Anxiety Scale (HAMA; Hamilton, 1959) 1 month after the stroke. The neurologists who assessed sleep quality and the psychiatrists who assessed emotion worked independently and did not know the results of the others' evaluations.

### 2.3 Definition of groups

Pittsburgh Sleep Quality Index is a self‐report questionnaire for the subjective measurement of an adult's sleep quality during a period of 1 month (Buysse et al., 1989). According to other studies, a score of 7 means good sleep, 7–11 indicates mild sleep disorder, 12–16 indicates moderate sleep, and 17–21 means severe sleep disorder (Wang, Wang, et al., 2019; Zhang et al., 2014). Based on their PSQI score at admission, the patients were divided into a poor sleep quality group (PSQI > 7, *n* = 76) and a good sleep quality group (PSQI ≤ 7, *n* = 251).

### 2.4 Assessment of post‐stroke anxiety

One month after the stroke, the patients were screened for anxiety symptoms using the 17‐item Hamilton Anxiety Scale (HAM‐A). The patients with anxiety symptoms, that is, a HAM‐A score >7 were diagnosed with PSA (Wu et al., 2016).

### 2.5 Statistical analyses

All continuous variables were expressed as the mean ± standard deviation (*SD*). The categorical variables were presented as frequencies and percentages. The different groups of normally distributed variables were compared using Student's *t* test or analysis of variance (ANOVA). The Mann–Whitney *U* test was applied to the non‐normally distributed variables. The chi‐squared test was employed to compare the categorical variables. Differences in two‐group comparisons were evaluated using the post hoc LSD test. The adjustment of multiple testing in each test was achieved by Bonferroni corrections. The independent risk factors of PSA were analyzed using the multivariate‐adjusted logistic regression that calculates odds ratios (ORs) and the corresponding 95% confidence intervals (CIs). All statistical analyses were conducted using SPSS for Windows, version 22.0 (SPSS Inc.). *p* < .05 was considered as statistically significant.

## 3 RESULTS

### 3.1 Baseline characteristics of patients in the two sleep quality groups

A total of 327 patients with AIS were enrolled for this study and followed up for 1 month. To compare the baseline characteristics (Table 1), the 327 patients were divided into the poor sleep quality group (*n* = 76) and the good sleep quality group (*n* = 251). There was no difference in demographic and cerebrovascular risk factors between the patients with the different sleep qualities (Table 1). The poor sleep quality patients had a higher rate of vitamin D deficiency and PSA than those with good sleep quality (36.8% vs. 14.7%, *p* < .001; 42.1% vs. 21.9%, *p* = .001).

**TABLE 1:** Baseline characteristics of patients in poor sleep quality and good sleep quality patients

Variables	Poor sleep quality (*n* = 76)	Good sleep quality (*n* = 251)	*p*‐value
Demographic characteristics			
Age (year)	62.33 ± 11.6	60.96 ± 11.1	.351
Gender, female, *n* (%)	32 (42.1%)	88 (35.1%)	.279
Education, *n* (%)			
None	29 (38.2%)	75 (29.9%)	.318
Primary	24 (31.6%）	89 (35.5%）	
Secondary	20 (26.3%)	64 (25.5%)	
Higher	3 (3.9%)	23 (9.2%)	
Income, *n* (%)			
Low	24 (31.6%)	71 (28.3%)	.246
Moderate	42 (55.3%)	125 (49.8%)	
High	10 (13.2%)	55 (21.9%)	
Marital status, Married, *n* (%)	62 (81.6%)	222 (88.4%)	.121
Vascular risk factors, *n* (%)			
History of hypertension	58 (76.3%)	182 (72.5%)	.511
History of diabetes mellitus	16 (21.1%)	65 (25.9%)	.391
Coronary artery disease	5 (6.6%)	8 (3.2%)	.185
Smoking			
Never	41 (53.9%)	136 (54.2%)	.803
Former	14 (18.4%)	39 (15.5%)	
Current	21 (27.6%)	75 (30.3%)	
Current drinking	28 (36.8%)	99 (39.4%)	.788
Laboratory parameters			
Vitamin D deficiency, *n* (%)	28 (36.8%)	37 (14.7%)	<.001
HbA1c (%)	6.2 ± 1.2	6.5 ± 1.7	.170
Clinical characteristics			
NIHSS score	3.0 (1.0–4.8)	2.0 (1.10–4.0)	.552
Poor outcome, *n* (%)	27 (35.5%)	72 (28.7%)	.258
MMSE score	24.0 (20.0–27.0)	23.0 (18.0–26.0)	.046
PSA, *n* (%)	32 (42.1%)	55 (21.9%)	.001

Abbreviations: MMSE, Mini‐Mental State Examination; NIHSS, National Institutes of Health Stroke Scale; PSA, post‐stroke anxiety.

### 3.2 Baseline characteristics of patients in PSA group and Non‐PSA group

Eighty‐seven (26.6%) patients showed symptoms of anxiety 1 month after the stroke. Compared with the stoke patients without PSA, we found that the patients with PSA were mostly females (46% vs. 33.3%, *p* = .039), had poor sleep quality (35.6% vs. 18.8%, *p* = .001), a lower rate of marriage (77.5% vs. 90.4%, *p* = .007), a higher percentage of moderate to low income (*p* < .005), vitamin D deficiency (29.9% vs. 15.4%, *p* = .006), more severe stroke (NIHSS score, 3.0 (2.0–6.0) vs 2.0 (1.0–4.0), *p* = .008), and poorer daily activities (41.1% vs 26.2%, *p* = .009, Table 2).

**TABLE 2:** Baseline characteristics of patients in PSA and non‐PSA patients

Variables	PSA (*n* = 87)	Non‐PSA (*n* = 240)	*p*‐value
Demographic characteristics			
Age (year)	60.6 ± 12.2	61.5 ± 10.9	.541
Gender, female, *n* (%)	40 (46%)	80 (33.3%)	.039
Education			
None	23 (26.4%)	81(33.75%)	.144
Primary	29 (33.3%)	84 (35%)	
Secondary	30 (34.5%)	54 (22.5%)	
Higher	5 (5.7%)	21 (8.75%)	
Income			
Low	34 (39.1%)	61 (25.4%)	.015
Moderate	43 (49.4%)	124 (51.7%)	
High	10 (11.5%)	55 (22.9%)	
Marital status, Married, *n* (%)	69 (77.5%)	217 (90.4%)	.007
Vascular risk factors, *n* (%)			
History of hypertension	64 (73.6%)	176 (73.3%)	.544
History of diabetes mellitus	22 (25.3%)	59 (24.6%)	.886
Coronary artery disease	7 (8%)	7 (2.9%)	.060
Smoking			
Never	52 (59.8%)	125 (52.1%)	.454
Former	13 (14.9%)	40 (16.7%)	
Current	22 (25.3%)	75 (31.2%)	
Current drinking	30 (34.5%)	97 (40.4%)	.331
Laboratory parameters			
Vitamin D deficiency, *n* (%)	26 (29.9%)	39 (15.4%)	.006
HbA1c (%)	6.3 ± 1.4	6.5 ± 1.7	.318
Clinical characteristics			
NIHSS score	3.0 (2.0–6.0)	2.0 (1.0–4.0)	.008
Poor outcome, *n* (%)	36 (41.1%)	63 (26.2%)	.009
MMSE score	22.0 (17.0–26.0)	24.0 (19.0–27.0)	.063
Poor sleep quality, *n* (%)	31 (35.6%)	45 (18.8%)	.001

Abbreviations: MMSE, Mini‐Mental State Examination; NIHSS, National Institutes of Health Stroke Scale; PSA, post‐stroke anxiety.

### 3.3 Association between sleep quality and PSA

Univariate logistic regression analysis showed that 1 month after stroke, poor sleep quality, high NIHSS scores, moderate and low income levels, and vitamin D deficiency were associated with PSA (OR: 2.265, 95% CI: 1.262–4.067, *p* = .003; OR: 1.153, 95% CI: 1.024–1.297, *p* = .018; OR: 2.330, 95% CI: 1.032–5.259, *p* = .042; OR: 3.101, 95% CI: 1.317–7.300, *p* = .010; OR: 3.000, 95% CI: 1.582–5.638, *p* = .001, respectively; Table 3). After adjusting for these confounding variables, poor sleep quality was still independently correlated with PSA (OR: 2.676, 95% CI: 1.451–4.936, *p* = .001). Interestingly, we found that income was correlated with PSA. The adjusted OR for PSA was 2.617 in the moderate‐income patients versus high‐income patients. A similar but significant difference was also found between patients in the low‐income versus the high‐income categories (OR: 3.980; 95% CI: 1.603–9.881; *p* = .003; Table 3).

**TABLE 3:** Multivariate logistic regression analysis for risk factors of PSA

Variable	Unadjusted			Adjusted model		
	OR	95% CI	*p*‐value	OR	95% CI	*p*‐value
Age	0.993	0.972–1.015	.540	0.982	0.959–1.106	.138
Gender, female	1.297	0.746–2.253	.356	1.560	0.864–2.817	.140
Education level (years)	1.014	0.953–1.079	.664	1.075	0.992–1.166	.079
Married	0.678	0.319–1.439	.311	0.521	0.236–1.149	.106
Income						
Moderate versus high	2.330	1.032–5.259	.042	2.617	1.135–6.032	.024
Low versus high	3.101	1.317–7.300	.010	3.980	1.603–9.881	.003
Vitamin D deficiency	3.000	1.582–5.638	.001	2.963	1.536–5.715	.001
NIHSS score	1.153	1.024–1.297	.018	1.150	1.022–1.295	.021
Poor sleep quality	2.265	1.262–4.067	.003	2.676	1.451–4.936	.001
MMSE score	0.961	0.921–1.103	.069	0.969	0.924–1.017	.203
Poor outcome	1.529	0.815–2.868	.186	1.419	0.739–2.724	.293

Abbreviations: MMSE, Mini‐Mental State Examination; NIHSS, National Institutes of Health Stroke Scale; PSA, post‐stroke anxiety.

## 4 DISCUSSION

As far as we know, this study is the first one to explore the association between sleep quality and PSA. Our results demonstrate that poor sleep quality before AIS is a significant risk factor for anxiety in AIS patients 1 month after the stroke onset, regardless of whether or not there was a history of sleep disorders before the stroke.

Sleep is of vital importance for health (Luyster, Strollo, Zee, & Walsh, 2012). Sleep deprivation can exert harmful effects on mental health (Roberts, Roberts, & Duong, 2009). As a response to mild stressors, sleep deprivation could create escalated negative effects on people (Minkel et al., 2012). Individuals who suffer from lack of sleep are more prone to develop anxiety and depression (Choueiry et al., 2016; Luyster et al., 2012). Numerous studies have confirmed the bidirectional association between insomnia and anxiety (Alvaro et al., 2013). Sleeping problems are a vital manifestation of anxiety and depression (Kokras et al., 2011). Previous studies have shown that insomnia or poor sleep quality is an important precursor and indicator of anxiety (Neckelmann et al., 2007; Sørengaard et al., 2019; Vedaa et al., 2016). These studies have highlighted the correlation between insomnia or poor sleep quality and anxiety. The results of the present study indicate that poor sleep quality is a significant risk factor for anxiety in AIS patients 1 month after the stroke onset. As a common and long‐lasting complication, early recognition and treatment are particularly important, but the mechanism of PSA induced by poor sleep quality remains unclear. Currently, some previous studies have shown that sleep deprivation leads to impaired executive functioning (Drummond, Gillin, & Brown, 2001; Durmer & Dinges, 2005; Nilsson et al., 2005), including the inhibition of attention and memory functions (Drummond et al., 2001; Durmer & Dinges, 2005). Reduced functional connectivity with the prefrontal cortex may reduce connections to brain areas associated with executive functions, thus reducing the brain's capacity to regulate and inhibit anxiety (Cox & Olatunji, 2016; Ma, Dinges, Basner, & Rao, 2015; Verweij et al., 2014; Wright et al., 2015) Disorder of the HPA axis is also associated with anxiety‐related disorders. Sleep deprivation increases the secretion of cortisol in human body (Wright et al., 2015). Specifically, the decrease of sleep time is related to the gradual decline of cortisol (Van Lenten & Doane, 2016) and the increase of cortisol secretion at night (Abell, Shipley, Ferrie, Kivimäki, & Kumari, 2016). The HPA axis abnormality is also evident in anxiety disorders. For example, cortisol output is reduced in patients with PTSD (Morris, Compas, & Garber, 2012), and in women with anxiety disorders, cortisol responses are insensitive when facing acute stressors (Zorn et al., 2017). Over time, chronically increases in cortisol or an inadequate response to acute stress may influence the occurrence of anxiety disorders (Cox & Olatunji, 2020). Moreover, most sleep regulation models involved the monoamine and cholinergic systems, and the inhibitory GABA (γ‐aminobutyric acid) mechanisms in sleep regulation (Mignot, Taheri, & Nishino, 2002). Because the dysfunction of these neurotransmitter systems is related to anxiety (Kent, Mathew, & Gorman, 2002), the changes of GABA caused by sleep disorders may mediate the occurrence of anxiety disorders (Staner, 2003).

A study from the South London Stroke Register indicated that in long‐term observations, PSA was a common problem, with the 10‐year prevalence rate ranging from 17% to 24% and a cumulative incidence of 57% (Ayerbe et al., 2014). A meta‐analysis of 44 studies comprising 5,760 stroke patients reported a pooled PSA prevalence of 20% 1 month after the stroke onset (Campbell Burton et al., 2013), which is similar to our results. We found that the patients with higher NIHSS scores were more likely to develop PSA than those with lower NIHSS scores. Stroke severity and physical disability have been reported to be predictors of PSA (Castillo et al., 1995), consistent with the findings of our study. Using multiple stepwise logistic regression analysis, we found that lower serum 25‐hydroxyvitamin D [25(OH)D] level (≤38.48 nmol/L) was also a risk factor of PSA, which is in accordance with the results of our previous study (Wu et al., 2016). The present study demonstrated females and patients with poor functional outcome tended to have a higher prevalence of PSA. Nevertheless, in multiple stepwise logistic regression analysis, no association was found between these two factors and PSA, which may be attributed to the relatively small number of females and patients with poor functional outcome enrolled in this study. Furthermore, we found moderate and low incomes were correlated with PSA. Few previous studies have focused on the relationship between annual income and PSA. A study of anxiety in gynecologic cancer patients showed that low household income was associated with anxiety (Corrales et al., 2018). A cross‐sectional observational study in China pointed out that the factors associated with poor concordance rate included the patient's annual household income, and clinically significant self‐reported symptoms of anxiety and hypochondriasis (Wang, Murray, et al., 2019). The MMSE score and educational level were not associated with PSA, which needs to be validated through further studies.

This study has several limitations: First, the relatively small sample size weakened the statistical strength of the study. Thus, a multicenter study with a larger sample size is needed. Second, a 1‐month (rather than 3 or 6 months) period was used in PSA assessment. It could be more useful to evaluate “true” anxiety rather than “reactive” anxiety. Third, the short follow‐up precluded us from exploring the effects of lengthy institutionalization on PSA. Moreover, severe aphasia patients were not included in the study due to their inability to complete the assessment, which might have weakened the generalization of the present study. Fourth, baseline levels of anxiety were not controlled. Fifth, although the PSQI is a measure of sleep over the past month, retrospective bias may increase the reporting of sleep disturbance following stroke. Finally, the data of depression symptoms were not available.

## 5 CONCLUSIONS

This study demonstrates that poor sleep quality before stroke is associated with PSA and may be an independent risk factor of PSA 1 month after the stroke onset. Hopefully, our findings would contribute to the prevention and treatment of PSA.

Our findings suggest that for patients with AIS, attention should be paid to the screening of sleep quality level at admission, and for patients with poor sleep quality, the assessment of anxiety level should be strengthened for early intervention. Further, multicenter and larger prospective studies are needed to confirm this association.

## CONFLICT OF INTEREST

None of the authors has any conflict of interest to disclose.

## AUTHORS' CONTRIBUTIONS

Xiao MJ and He JC designed the study. Xiao MJ and Huang GQ interpreted data. Feng L, Luan XQ, Wang QZ, and Ren WW prepared figures. Huang GQ, Xiao MJ, and Feng L did the statistical analyses. Huang GQ, Luan XQ, Wang QZ, and Chen SY screened and extracted data. He JC supervised study. All authors have made an intellectual contribution to the manuscript and approved the submission.

## Data Availability

The data that support the findings of this study are available on request from the corresponding author. The data are not publicly available due to privacy or ethical restrictions.
